# Nephrotic syndrome in a dish: recent developments in modeling in vitro

**DOI:** 10.1007/s00467-019-4203-8

**Published:** 2019-02-28

**Authors:** Susan Veissi, Bart Smeets, Lambertus P. van den Heuvel, Michiel F. Schreuder, Jitske Jansen

**Affiliations:** 1 Department of Pediatric Nephrology, Radboud Institute for Molecular Life Sciences, Amalia Children’s Hospital, Radboud University Medical Center, Nijmegen, The Netherlands; 2 Department of Pathology, Radboud Institute for Molecular Life Sciences, Radboud University Medical Center, Nijmegen, The Netherlands; 3 Department of Laboratory Medicine, Radboud Institute for Molecular Life Sciences, Radboud University Medical Center, Nijmegen, The Netherlands; 4 Department of Development and Regeneration, University Hospital Leuven, Leuven, Belgium

**Keywords:** Kidney, Nephrotic syndrome, Glomerular filtration barrier, Podocytes, Stem cells, Organoids

## Abstract

Nephrotic syndrome is a heterogeneous disease, and one of the most frequent glomerular disorders among children. Depending on the etiology, it may result in end-stage renal disease and the need for renal replacement therapy. A dysfunctional glomerular filtration barrier, comprising of endothelial cells, the glomerular basement membrane and podocytes, characterizes nephrotic syndrome. Podocytes are often the primary target cells in the pathogenesis, in which not only the podocyte function but also their crosstalk with other glomerular cell types can be disturbed due to a myriad of factors. The pathophysiology of nephrotic syndrome is highly complex and studying molecular mechanisms in vitro requires state-of-the-art cell-based models resembling the in vivo situation and preferably a fully functional glomerular filtration barrier. Current advances in stem cell biology and microfluidic platforms have heralded a new era of three-dimensional (3D) cultures that might have the potential to recapitulate the glomerular filtration barrier in vitro. Here, we highlight the molecular basis of nephrotic syndrome and discuss requirements to accurately study nephrotic syndrome in vitro, including an overview of specific podocyte markers, cutting-edge stem cell organoids, and the implementation of microfluidic platforms. The development of (patho) physiologically relevant glomerular models will accelerate the identification of molecular targets involved in nephrotic syndrome and may be the harbinger of a new era of therapeutic avenues.

**Electronic supplementary material:**

The online version of this article (10.1007/s00467-019-4203-8) contains supplementary material, which is available to authorized users.

## Introduction

Nephrotic syndrome (NS) is one of the most common causes of glomerular disease in childhood with an estimated incidence of 1–7 per 100,000 children [1, 2]. NS is characterized by the triad of proteinuria (> 40 mg/m^2^/h), hypoalbuminemia (< 2.5 g/dL), and edema [2]. Different etiologies may lead to NS. Congenital NS is a rare and severe form of NS, which occurs due to genetic mutations and secondary to congenital infections. Acquired NS is mostly idiopathic (iNS), but can also be acquired secondary to infections, drugs, or neoplasia [2]. Depending on its etiology, NS may resolve over time or result in a gradual loss of kidney function, finally leading to the need for dialysis or a kidney transplant.

**Fig. 1:**
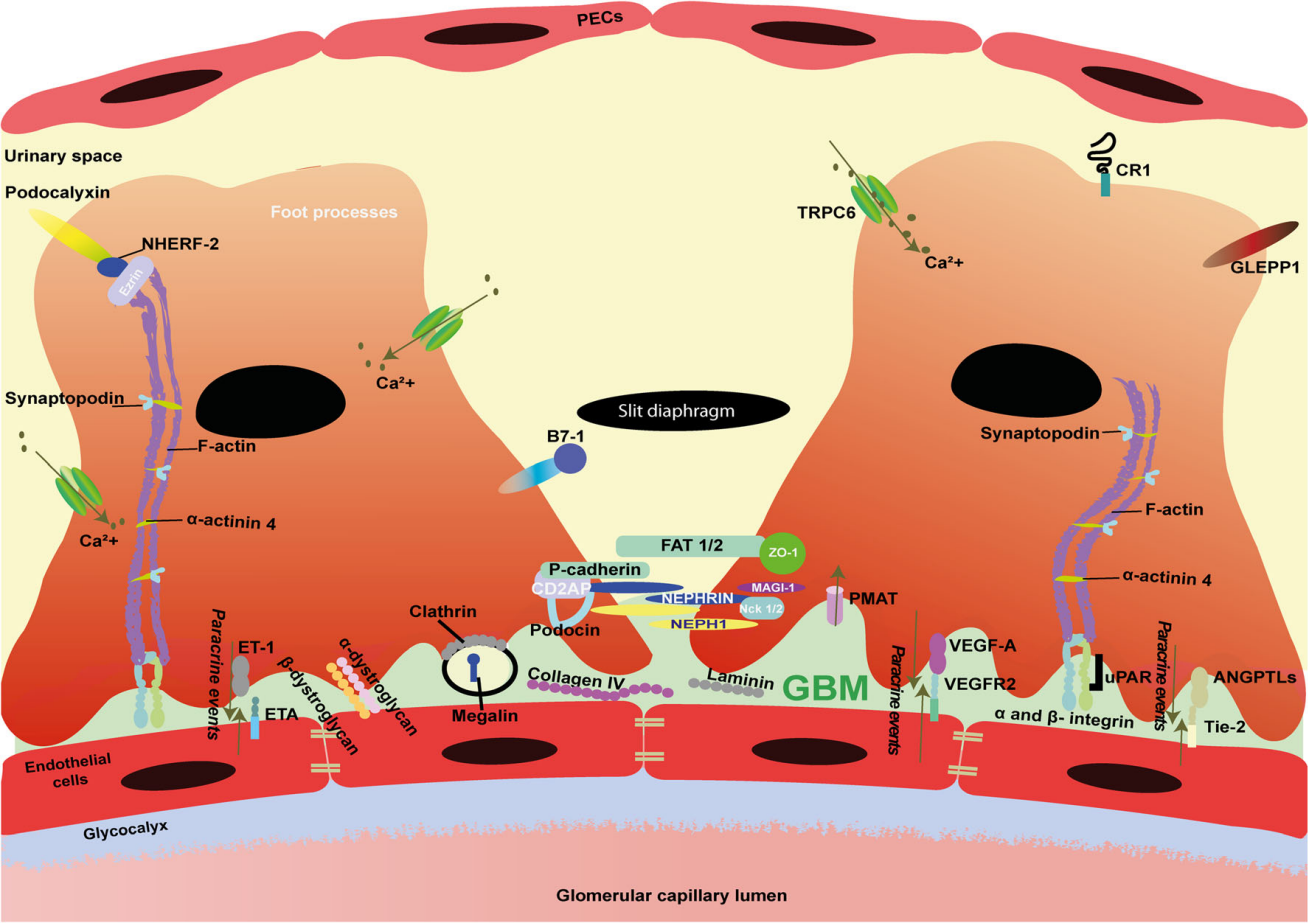
Schematic illustration depicting glomerular filtration barrier and molecular overview of podocytes. The three-layered glomerular filtration barrier, consisting of fenestrated endothelial cells, glomerular basement membrane (GBM), and podocytes. Molecules depicted are relevant for the function and characterization of podocytes. These molecules include slit diaphragm, cytoskeleton, and foot processes molecules. Furthermore, hypothesized paracrine signaling molecules (ANGPTLs, VEGF-A, and ET-1) and their receptors, Tie-2, VEGFR2, and ETA, respectively, that are responsible for the crosstalk between endothelial cells and podocytes are shown as well. Finally, podocyte injury markers, such as GLEPP-1, B7-1, CR1, and ezrin are included in the illustration. GBM, glomerular basement membrane; PECs, parietal epithelial cells; ANGPTLs, angiopoietins; CD2AP, CD2-associated protein; MAGI1, membrane-associated guanylate kinase; Nck1/2, non-catalytic region of tyrosine kinase adaptor protein 1/2; NEPH1, same as kin of IRRE-like protein 1 (KIRREL); NPHS1, nephrin; PMAT, plasma membrane monoamine transporter; TRPC6, Transient Receptor Potential Cation Channel Subfamily C Member 6; NHERF-2, Sodium-hydrogen exchange regulatory cofactor NHE-RF2; GLEPP-1, glomerular epithelial protein 1; VEGF-A, vascular endothelial growth factor A; VEGFR2, vascular endothelial growth factor A; ZO-1, zonula occludens-1; α-ACTN4, alpha actinin 4; Ca^2+^, calcium; ET-1, endothelin 1; ETA, endothelial ET-1 receptor A; Tie-2, tyrosine kinase receptor 2; uPAR, urokinase plasminogen activator receptor

From a molecular point of view, NS is the result of an altered glomerular filtration barrier, which is a trilayered structure consisting of fenestrated (glycocalyx-covered) endothelium, the glomerular basement membrane (GBM), and polarized visceral epithelial cells known as podocytes (Fig. 1). Podocytes are epithelial cells with interdigitating foot processes that are connected by cell-cell junctions known as slit diaphragms. Podocytes possess an extensive actin cytoskeleton, which aids the glomerular capillary wall to withstand the high hydrostatic pressure required for glomerular filtration [3]. In Supplementary Table S1, key proteins involved in maintaining a well-organized podocyte cytoskeleton are listed along with their physiological characteristics.

Podocyte damage is considered a hallmark event in the pathogenesis of NS. As these cells are endowed with a limited capacity to regenerate, loss of podocytes or podocyte damage is hard to restore. In Supplementary Table S1, injury markers used to detect podocyte damage are mentioned. For example, desmin is de novo expressed during podocyte injury and thus reflects a typical injury marker [4]. Concomitantly, damage to podocytes is characterized by podocyte foot process effacement and structural changes to the slit diaphragms as well as the actin cytoskeleton [2]. Regardless of the fact that podocytes are considered the main player in NS pathophysiology, it is important to stress that NS should be considered as a disorder of the entire glomerulus. Hence, degradation of the glycocalyx, injury to glomerular endothelial cells, or alterations in the GBM can also cause protein leakage and contribute to disease [5].

Understanding the pathophysiological processes involved in NS is a prerequisite to develop targeted therapies. As such, in vivo and in vitro models are important tools to unravel molecular mechanisms involved in NS and can eventually be used to screen for pharmacological compounds. In vivo models to study congenital and acquired NS have greatly expanded our knowledge on NS pathophysiology [6, 7]. As a result of whole exome sequencing, a myriad of unknown genetic variants are detected in congenital NS patients nowadays. Unfortunately, the sheer number of variants is too numerous to model all in vivo. Moreover, species differences may hamper the translation from mice to men. In addition, the paradigm shift in animal reduction, refinement, and replacement urges the need for appropriate in vitro models to mimic NS in a dish. Hence, accurate and personalized in vitro models are highly needed in the NS field. In vitro podocyte cultures have proven to be an indispensable tool to assess functionality of these cells [8, 9]. Although current in vitro models have yielded valuable insight into molecular mechanisms of podocytes in health and disease, these models fail to fully recapitulate the complex biology of podocytes for several reasons. First, current podocyte in vitro models contain only one cell type, second, the podocytes are grown in a two-dimensional (2D) setting and third, the podocytes in culture are not exposed to a blood stream and thus not exposed to intracapillary pressure and glomerular filtration. With the rapid evolvements in the field of stem cell biology and bioengineering, novel advanced platforms to study the complex pathology of NS in vitro can be expected to revolutionize the renal field within the near future. Therefore, the aim of this review is to address recent developments regarding the pathophysiology of NS and discuss current and novel in vitro cell models to study NS.

## Current models to study NS pathophysiology in vitro

To date, the pathogenic events leading to an altered glomerular filtration barrier in NS are still largely unknown; thus, so far, the involvement of genetic mutations, T- and B-lymphocytes, circulating (permeability) factors (CPFs), and paracrine events between podocytes and endothelial cells have been suggested [2]. The complexity of NS makes it challenging to study its pathophysiology accurately, which is further hampered by the poor recapitulation of the glomerular filtration barrier by the frequently used in vitro models. Here, we will discuss current knowledge on NS pathophysiology and in vitro models that have been used so far to study the molecular mechanism of disease.

### Functional assessment of genetic variants

Various genetic mutations that lead to podocyte abnormalities have been identified as causative for familial forms of both NS and iNS. The genetic defects that underlie NS, in particularly steroid-resistant NS (SRNS), are mainly mutations in a single gene causing structural (i.e., changes in the slit diaphragms and actin cytoskeleton) or functional defects, affecting, for example, mitochondrial and lysosomal proteins or transcription factors in podocytes. Owing to the rapid advancement of whole exome sequencing (WES), single-gene mutations in at least 53 genes have been identified as causative for NS (for an overview, please see [10]). To study the functional consequences of genetic variants detected by WES, accurate in vitro assays are required. Human embryonic kidney (HEK) cells are easy to transfect and allow the study of the role of mutations in terms of expression levels, post-translational modifications, and cell-surface trafficking [11, 12]. However, HEK cells poorly recapitulate the complex podocyte biology as mature podocytes are characterized by an arborized phenotype, expression of focal adhesion complex proteins, slit diaphragm junctions, and processes like receptor-mediated endocytosis and calcium flux. Although human conditionally immortalized podocytes have been used to study podocin mutations [13], immortalized cells have limited podocyte characteristics [14, 15], warranting more optimal models. Primary podocyte cultures, freshly isolated from rodent or human glomeruli, most closely resemble the in vivo situation, but a rapid dedifferentiation and a limited proliferation capacity hinders the use of primary cells in the research setting [16]. During the past decades, isolation and culture protocols have been further optimized to stimulate podocyte-specific characteristics and the reproducibility of primary cultures [17]. Nowadays, murine models with genetically tagged podocytes are available that allow podocytes to be specifically isolated from other glomerular cells [18]. Yang et al. recently showed that fluid sheer stress and retinoic acid supplementation induced synaptopodin, podocin, WT-1, and ZO-1 expression in human primary podocytes [18, 19]. Still, donor variability and the quiescent properties of (partially) differentiated primary cultures challenge the use of primary cells in vitro. Alternatively, renal progenitor cells have been isolated from urine of NS children diagnosed with mutations in NPHS2 and LMX1b [20]. The progenitor cells were differentiated in vitro towards podocyte-like cells and functional testing showed a clear reduction in NPHS2 expression and a deteriorated cytoskeleton architecture, while the mutated transcription factor LMX1b was associated with altered podocin localization as well as a disturbed cytoskeleton [20]. Urinary renal progenitor cells have the potential to be used as a non-invasive source for studying personalized genetic kidney disease, but the number of progenitor cells present in urine varies and hampers the success rate of urine-derived progenitor and associated podocyte cultures. To overcome this hurdle, the use of (patient-derived) induced pluripotent stem cells (iPSC) and differentiation towards podocytes is a promising approach [21–24]. Tanigawa and co-workers successfully reprogrammed skin fibroblasts isolated from a newborn diagnosed with congenital NS into iPSC [12]. This work nicely showed reduced nephrin expression and cell surface localization, due to disturbed protein glycosylation, and impaired slit diaphragm formation in iPSC-derived kidney organoids containing podocytes. In contrast to primary or immortalized podocyte models that either rapidly dedifferentiate or have poor podocyte characteristics (e.g., lack of slit diaphragm), iPSC kidney organoids do express podocyte-specific markers (e.g., nephrin, podocin) and proteins are well localized. Hence, kidney organoids are promising tools for studying personalized congenital NS and have the potential to unravel molecular mechanisms involved in its pathogenesis. In the final section of this review, the current status of iPSC differentiation towards podocyte-like cells and kidney organoids will be discussed.

### Immune factors involved in NS

It has been recognized that lymphocytes play a role in the pathophysiology of NS. Initial evidence comes from measles infection, which results in repressed cell-mediated immunity and consequently induced remission in two pediatric NS patients [2, 25]. The fact that immunosuppressive agents that particularly inhibit T cell functions exert therapeutic effect in NS patients supports this. Moreover, chemotherapy treatment of T cell lymphomas (i.e., Hodgkin’s), which can be a trigger or precede NS, resulted in spontaneous recovery, further supporting this hypothesis [26]. Alterations in T cell subsets, particularly an increase in CD4^+^ T-helper (Th) cells and a (concurrent) decline in regulatory T cells, have been described in NS patients [27]. To date, mainly Th cell subsets Th2 and Th17 are associated with NS [28, 29]. The upregulation of a specific cytokine profile (interleukin (IL) 4 (IL4), IL5, IL9, IL10, IL13) associated with Th2 cells was previously shown to promote the development of NS [28]. In addition, it was reported that the production of IL17 may contribute to podocyte injury by decreasing podocalyxin levels resulting in proteinuria and podocyte loss [29]. Altogether, these data suggest that T cells play an important role in the pathophysiology of NS. A role for B cells in NS is underscored by the beneficial effects of rituximab (a neutralizing antibody against B cells) in steroid-dependent NS patients [30]. To date, no accurate in vitro assays have been successfully developed to study the role of lymphocytes in NS. The complexity of developing a robust co-culture that consists of specific lymphocytes and podocytes likely adds to this gap. Recent developments in the field of organs-on-a-chip could reveal opportunities to better mimic the renal microenvironment including the immune system. The current state of organs-on-a-chip will be addressed in the final section of this review.

### Evidence of circulating permeability factors

In 1954, the presence of circulating permeability factors (CPFs) causing structural changes to the glomerular filtration barrier in NS was suggested by Gentili et al. [31]. This suggestion was based on an experiment in which plasma of an infant with NS was given to a non-nephrotic child, who subsequently developed massive proteinuria [31]. Further evidence for the role of CPFs comes from the recurrence of Focal segmental glomerulosclerosis (FSGS) after kidney transplantation, as well as the decreased levels of proteinuria in such patients who are treated with plasmapheresis compared to those not treated with plasmapheresis [32, 33]. The most compelling evidence for a CPF arose from a case report, in which a renal allograft that was removed 14 days post-transplantation due to the recurrence of FSGS was retransplanted to another patient (with type 2 diabetes) after which it regained its function and did not show proteinuria [34]. The source of CPFs (e.g., that they originate from B or T cells) has been much speculated on, including the actual form of these CPFs. Such options include circulating immune complexes, factors associated with infections, or toxins. Specifically, inflammatory mediators or host-derived molecules, such as soluble urokinase plasminogen activator receptor (suPAR), hemopexin, and cardiotrophin-like cytokine 1 (CLC-1), have been suggested as CPF [35].

Cultured podocytes are frequently used to investigate the presence and toxicity of CPFs in vitro [13, 36–38]. To investigate the effect of a putative CPF, cells are exposed to plasma from patients with primary or recurrent FSGS and toxicity effects on the podocyte cytoskeleton and motility characteristics are studied using a scratch assay. Kachurina and co-workers developed a method to quantitatively measure focal adhesion complex density, based on vinculin expression and distribution [37, 38]. This unbiased method circumvents qualitative analysis of cytoskeleton integrity as performed in the past [36]. Motility is a known podocyte characteristic coordinated by both microtubules and actin. However, the degree of podocyte motility to be considered normal or pathological remains still elusive [39]. It was shown that enhanced migration correlates with induced proteinuria suggesting that motility is involved in the pathological events of NS [13]. Harris et al. showed that podocyte migration was induced when cells were exposed to plasma samples collected from patients undergoing the primary plasmapheresis session for FSGS relapse following kidney transplantation [13]. Concomitantly, Harris et al. identified that proteases present in relapse plasma activate protease activate receptor-1, resulting in podocin-dependent phosphorylation of vasodilator stimulated phosphoprotein, in turn leading to enhanced motility [13]. Interactions between (s) uPAR and αVß3-integrin have also been associated with enhanced podocyte motility [40], although these findings could not be confirmed by others [41], and further investigation is required. In addition to immortalized podocytes, freshly isolated rat glomeruli have also been used to study the effects of CPFs [42]. The incubation of these rat glomeruli in recurrent FSGS patients’ serum resulted in reduced glomerular permeability and showed less glomerular swelling as compared to healthy controls’ serum. However, a lack of reproducibility of the method [43] hampers the use of this approach, thus data should be interpreted with care.

### Glomerular crosstalk in NS

An extensive body of evidence indicates that a hallmark of NS includes injury to podocytes, which is characterized by podocyte foot process effacement and structural changes to the slit diaphragm [2]. Consequently, podocytes have become the central focus of research in NS. However, studies from recent years have demonstrated endothelial cell involvement in the development of albuminuria [44, 45]. Therefore, an emerging concept of paracrine events, specifically the crosstalk between glomerular endothelial cells and podocytes, may be responsible for glomerular filtration barrier alterations in NS. This bidirectional paracrine network is illustrated by the action of signaling molecules such as vascular endothelial growth factor A (VEGF-A)/VEGF receptor-2 (VEGFR-2), angiopoietins (ANGPTs), endothelin-1 (ET-1)/ET-1 receptor A, and integrins (Fig. 1) [46–49].

Unfortunately, due to the lack of accurate cell co-culture models consisting of podocytes and endothelial cells, glomerular crosstalk has solely been studied in vivo*.* For example, deletion/knockout of podocyte VEGF-A or ANG-1 in mice has been shown to result in endothelial cell injury as well as increased glomerular damage [48, 50]. Furthermore, in vivo overexpression of podocyte VEGF-A seems to cause foot process effacement and proteinuria [48]. As shown by Eremina et al., VEGFR-2 knockout results in kidney abnormalities and vascular defects associated with glomerular thrombotic microangiopathy [51]. Additionally, in vivo overexpression of ANG-1 in podocytes results in reduced albuminuria and endothelial cell proliferation [50]. More recently, a hyposialylated form of angiopoietin-like 4 (ANGPTL4) produced by podocytes was shown to act on endothelial cells, leading to changes in the charge of the glomerular filtration barrier, potentially causing proteinuria [52]. Interestingly, a recent new crosstalk mechanism involving ET-1 has been revealed. ET-1 produced by endothelial cells was shown to activate podocytes to release heparanase, which is an enzyme that degrades polymeric heparan sulfate molecules present on endothelial cells or in the GBM, thereby causing proteinuria in mice [53]. Accurate in vitro co-culture models are warranted in order to recapitulate these in vivo findings. The co-culture models such as Transwell® system or organ-on-a-chip would more closely mimic the glomerular filtration barrier. Hence, these platforms would be highly valuable to accurately study aforementioned crosstalk events in vitro. These systems will be addressed in the final section of this review.

Over the past decades, a number of in vitro assays have been developed to study effects of genetic defects and permeability factors in NS. However, the cell models used exhibit poor podocyte characteristics that hamper accurate analysis of NS in a dish. As in vitro models representing the entire glomerular filtration barrier are in their infancy, it is even more complex to model the interactions between podocytes, the fenestrated endothelium and the GBM, which are affected in NS. As such, advanced cell-based models that better reflect the in vivo situation are a prerequisite to accurately study NS in vitro and will be addressed in the next section.

## Criteria for accurate in vitro models to study NS

Recent advances in the stem cell biology and bioengineering field have created opportunities for the development of potential platforms to study NS. Here, we will focus on the cutting-edge stem cell models and microfluidic culture systems that are revolutionizing the podocyte field.

### Modeling NS using stem cells

**Fig. 2:**
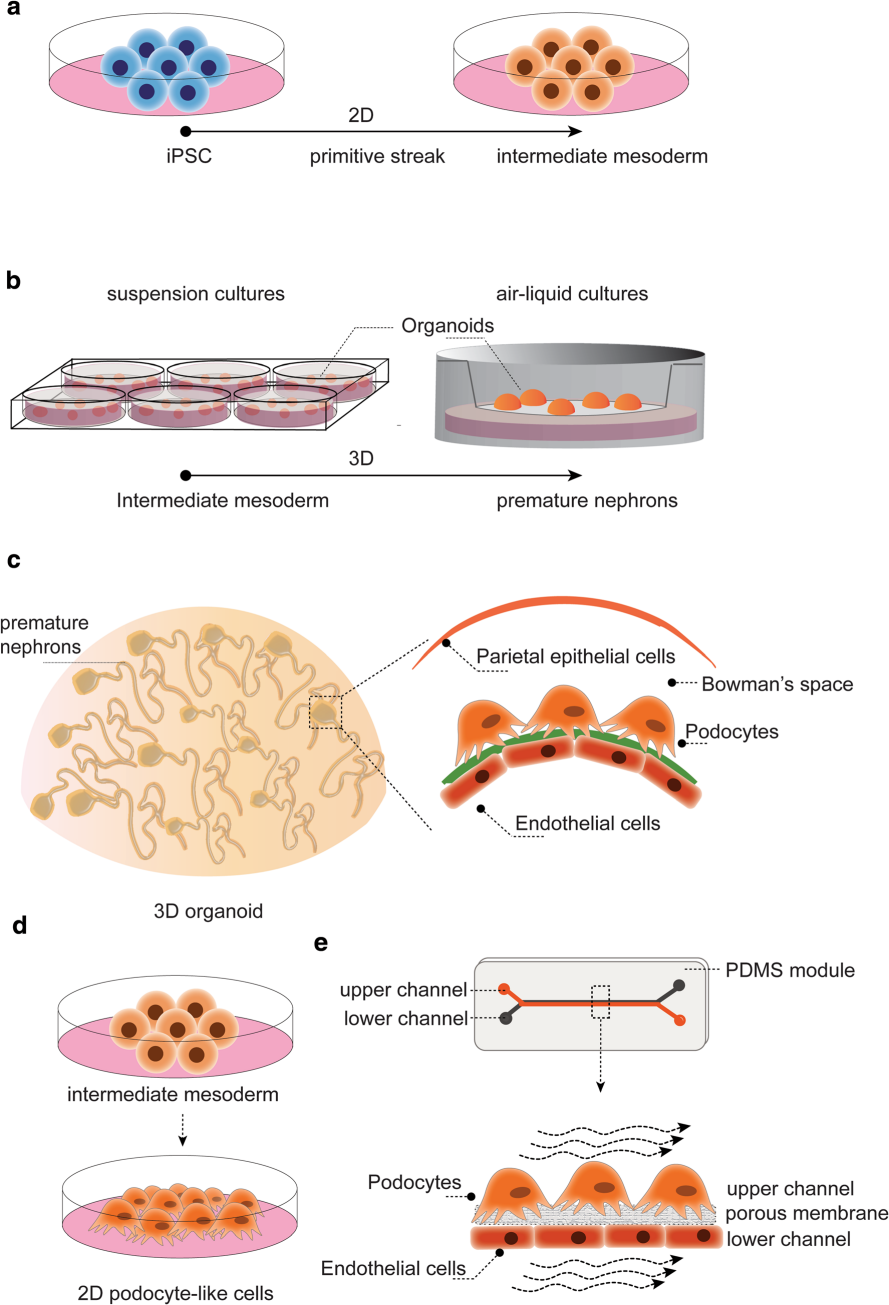
Schematic diagram of iPS cultures towards podocyte or organoid differentiation. **a** Differentiation from pluripotency towards intermediate mesoderm is usually performed in 2D. **b** Suspension and air-liquid cultures using Transwell® filter inserts to induce self-organizing kidney organoids. **c** Organoids contain multiple nephrons and glomeruli consist of podocytes, endothelial cells, glomerular basement membrane, and parietal epithelial cells, though not fully mature. **d** Differentiation of podocyte-like cells in 2D or **e** in a glomerulus-on-a-chip

Generating renal cells using human stem cells offers a promising approach to study podocytopathies. Amniotic fluid stem cells as well as kidney progenitor cells from neonatal urine have the potential to differentiate towards podocyte-like cells, though these sources are relatively scarce [54]. The recent progress of reprogramming any somatic cell type (e.g., human dermal fibroblasts) into pluripotent stem cells has opened a new dimension to develop, in principal, any desired cell type, including renal tissue. Using a directed differentiation protocol, embryonic kidney development is mimicked in a culture dish, from primitive streak, towards intermediate mesoderm followed by ureteric bud and metanephric mesenchyme induction. The latter combination is essential for bidirectional signal transduction that facilitates developmental processes, including the organotypic branched architecture of the kidney. To date, several protocols exist that generate diverse subsets of renal (progenitor) cells (Fig. 2). Using either 2D or 3D culture approaches, and varying the choice and/or timing of growth factors, result in the induction of one renal cell type (e.g., podocyte-like cells) [15, 21, 55, 56] or in self-organizing kidney organoids consisting of segmented nephrons [22, 23, 57–59]. These protocols are extensively reviewed by Soo et al. [60]. In brief, a combination of Wnt signaling agonist CHIR, fibroblast growth factors 2 and 9, bone morphogenic protein 2 or 4, as well as retinoic acid, vascular endothelial growth factor, and activin A are most commonly used as growth factors. The organoid differentiation protocols are executed in 2D using well plates, in 3D suspension cultures using low adhesion plates, or in 3D adherence cultures using the air-liquid interface of Transwell® filter inserts. While current protocols result in kidney organoids resembling first trimester kidney development and nephron progenitors [24, 61, 62], a robust protocol to generate a fully mature kidney-in-a-dish has not yet been established. In particular, glomerular microcirculation, peritubular vascularization, and flow throughout the entire organoid are lacking and hinder glomerular and tubular maturation in vitro. To push maturation, organoids were transplanted subcapsular in mouse kidneys, and massive graft vascularization was shown [59, 63, 64]. As a result, a fenestrated glomerular endothelium, a glomerular filtration barrier including GBM protein deposition and polarized podocytes, was observed. Moreover, this approach resulted in the successful analysis of congenital NS patient-derived organoids, and gene correction nicely restored nephrin expression in the podocyte slit diaphragm [12]. These data emphasize the importance of a functional microvasculature and accompanied hemodynamics to boost glomerular filtration barrier formation and kidney organoid maturation. The crosstalk between the glomerular endothelium and podocytes via the GBM is a known hallmark for developing and maintaining a filtration barrier function [65]. All taken together, 3D differentiation protocols resulting in self-organizing nephrons that contain subsets of renal epithelial and endothelial cells will more closely resemble the glomerular filtration barrier. Hence, organoids containing self-organizing nephrons could be a more relevant model to study NS, as compared to less complex 2D differentiation protocols that solely generate podocyte-like cells.

Although kidney organoid transplantation into murine models advances maturation, experiments are laborious, technically challenging, and expensive. While the use of kidney organoids for disease modeling or drug testing would potentially reduce animal testing, as urged by animal welfare committees as well as the social community, organoid transplantation would still require the use of animals.

Generating mature kidney organoids in vitro would be the preferred direction and it needs to be determined how far we can get. Hence, researchers are seeking cutting-edge approaches to stimulate maturation and develop robust screening platforms [22, 23, 57]. High-throughput analysis using 96- or 384-wells plate formats not only allows for the study of dose-dependent effects of, for example, growth factors on organoid differentiation, but also provides an ideal tool to systematically study drug responses and toxicity, as well as disease modeling [22, 57]. Recently, bulk organoid cultures have been generated using bioreactors [58]. This approach uses a simple 14-day method with few handling steps and results in embryonic kidney structures. A prolonged culture time results in dedifferentiation as determined by fibrotic markers [58]. Though this method potentiates the development of large amounts of kidney tissue, the differentiation capacity is not yet optimal. Borestrom et al. developed an advanced 3D differentiation protocol: organoids are submerged in medium using specific culture plates [57], rather than “standard” air-liquid Transwell® cultures [24]. This allows for the monitoring of maturation of progenitors and podocytes in a kidney-specific reporter model. Using a gene-editing system (CRISPR/Cas9), the kidney lineage markers SIX2 and NPHS1 were tagged with fluorescents. Monitoring the signal intensity of these two markers in live cells over time is a valuable indicator of nephron commitment (SIX2) and podocyte health (NPHS1) during maturation. The present protocol resulted in the formation of podocyte foot processes and early slit diaphragms in close proximity to endothelial and tubular cells [57]. Moreover, the data suggests infiltrating blood vessel-like structures (positive for the endothelial marker CD31) into glomerular structures [57]. Yet, an intact and functional glomerular filtration barrier still needs to be shown.

### Glomerulus-on-a-chip

Organoid vascularization is key in providing sufficient nutrients throughout the tissue and will push maturation, but also shear stress and hemodynamics are known factors that stimulate differentiation. Although some progress in terms of maturation has been made by slightly adapting timing, growth factors or culture platforms, the implementation of flow by microfluidic systems could further advance this field. In the past decade, significant progress has been made in generating microfluidic organs-on-a-chip that basically consist of cells cultured on a 2D surface or in 3D tubular structures accompanied by controlled fluid flow throughout the system, either luminal, basal or both. Proof-of-concept studies have showed the successful engineering of a glomerulus-on-a-chip [55, 66, 67] (Fig. 2). To recapitulate the podocyte-endothelial interface, the poly(dimethylsiloxane) (PDMS) chip consists of two microchannels; the top channel containing podocytes and the bottom channel containing glomerular endothelial cells separated by a porous extracellular matrix-coated membrane (made of, e.g., polycarbonate, PDMS). Musah et al. differentiated iPS cells in the chip using flow and cyclic mechanical strain. Interestingly, markers like nephrin expression and VEGF-A secretion were induced as compared to static cultures and no strain. Moreover, a functional glomerular filtration barrier was shown by albumin retention in the vascular channel, which could not be detected when proximal tubule cells were cultured in the top channel. Finally, drug-induced podocyte injury could be successfully mimicked [55]. It is noteworthy that 2D iPSC-podocyte differentiation did not result in the expected nuclear expression pattern of WT-1 and that the transcriptome profile of the iPS-podocytes was only compared with immortalized podocytes rather than using freshly isolated podocytes [15, 55]. The first steps towards hypertensive as well as diabetic nephropathy modeling in a glomerulus-on-a-chip have been initiated and show detrimental effects on, e.g., cytoskeleton rearrangements and protein expression levels [66, 67]. In these studies, either murine immortalized cells or human primary glomerular tissue were used. As both models poorly resemble podocyte (patho) physiology, data should be interpreted with care. The glomerulus-on-a-chip allows mimicking the microenvironmental cues required to model podocyte health and disease. Although the use of chips can be technically challenging and current protocols provide room for improvement, introducing shear stress and mechanical strains using microfluidic systems might push maturation in iPSC-derived kidney tissue.

## Concluding remarks

Studying molecular mechanisms involved in NS requires cutting-edge cell-based models to mimic the complex glomerular filtration barrier in vitro. Recent advances in stem cell biology and microfluidic platforms might overcome the lack of accurate models. Three-dimensional kidney organoids that recapitulate the glomerular filtration barrier could become a valuable tool to unravel NS-associated molecular mechanisms and identify therapeutic avenues. Advances to aid organoid vascularization and maturation, ultimately including a fluid inlet and urinary exit, will be a prerequisite to develop accurate platforms for studying both congenital and idiopathic NS.

## Electronic supplementary material

ESM 1 (DOCX 28 kb)
