# Supplementary material for: Nephrotic syndrome in a dish: recent developments in modeling in vitro
Source: Pediatr Nephrol. 2019 Feb 28;35(8):1363–72. doi: 10.1007/s00467-019-4203-8 (PMC7316697; doi:10.1007/s00467-019-4203-8)
Supplement: Supplementary file 1 — (DOCX 28 kb) [file 467_2019_4203_MOESM1_ESM.docx]

**Nephrotic syndrome in a dish; recent developments in modeling *in vitro*.**

*Supplemental material*

*Review Pediatric Nephrology*

Veissi S^1^, Smeets B^2^, van den Heuvel LP^1,3,4^, Schreuder MF^1^, Jansen J^1,2*^

^1^Radboud university medical center, Radboud Institute for Molecular Life Sciences, Amalia Children's Hospital, Department of Pediatric Nephrology, Nijmegen, The Netherlands.

^2^Department of Pathology, Radboud university medical center, Radboud Institute for Molecular Life Sciences, Nijmegen, The Netherlands.

^3^Department of Laboratory Medicine, Radboud university medical center, Radboud Institute for Molecular Life Sciences Nijmegen, The Netherlands.

^4^Department of Development and Regeneration, University Hospital Leuven, Leuven, Belgium.

^*^ Corresponding author

J. Jansen, PhD

Dept. of Pathology and Pediatric Nephrology

Radboud Institute for Molecular Life Sciences

Amalia Children’s Hospital

RadboudUMC

Geert Grooteplein 10, 6525 GA Nijmegen, The Netherlands

Phone: +31 (0) 243 66 820

Fax: +31 (0) 243 66 8750

E-mail: [jitske.jansen@radboudumc.nl](mailto:jitske.jansen@radboudumc.nl)Table 1: Podocyte and podocyte injury markers and assays to characterize each marker.

Table S1. Markers to study podocyte (patho)physiology

| Marker | Expression | Function | Ref. |
| --- | --- | --- | --- |
| Cell membrane markers | | | |
| Cortactin | Apical membrane | Assembly and maintenance of actin filaments network in the foot processes. | [1] |
| Megalin | Cell membrane | Endocytosis | [2, 3] |
| PMAT | Cell membrane | Organic cation transporter | [4] |
| Podocalyxin | Cytoskeleton (apical site above the slit diaphragm) | Maintenance of the foot processes structure. | [1, 5] |
| Podoplanin | Cytoskeleton (cell surface) | Maintenance of cell shape and foot process structure. | [6] |
| TRPC6 | Cell membrane | Control of Ca^2+^ -flux | [7] |
| Vimentin | Cytoskeleton and foot processes | Structural stability of cytoskeleton. | [8] |
| α/β-dystroglycan | Cell membrane (basal) | Maintenance of foot processes | [9] |
| α-ACTN4 | Cytoskeleton | Maintenance of the proper cytoskeleton and foot processes | [10] |
| Foot processes markers | | | |
| ANGPT1 | Foot processes | Maintenance of the glomerular permeability | [11, 12] |
| Myosin | Foot processes | Maintenance of actin cytoskeleton, endocytosis, and cell signalling | [13, 14] |
| Synaptopodin | Foot processes | Regulation of foot processes shape and motility. | [7] |
| T-/H-cadherin (CDH13) | Foot processes | Differentiation of podocytes and formation of glomerular capillary network. | [15] |
| VEGF | Foot processess | Endothelial fenestration | [11] |
| Nuclear markers | | | |
| WT-1 | Nuclear | Podocyte differentiation, epithelial-to-mesenchymal transition. | [16] |
| Slit diaphragm markers | | | |
| CD2AP | Slit diaphragm | An Adaptor for nephrin and podocin to anchor them to actin filaments of podocyte cytoskeleton. | [17] |
| FAT 1 | Slit diaphragm | Involved in cell-cell contact formation and integrity of slit diaphragms. | [18] |
| MAGI1 | Slit diaphragm | Involved in slit diaphragm formation. | [19] |
| Nck 1/2 | Slit diaphragm | An adaptor protein for nephrin involved in assembly and maintenance of actin cytoskeleton. | [20, 21] |
| NEPH1 | Slit diaphragm | Maintenance of glomerular permeability by binding to nephrin. | [22] |
| NPHS1 | Slit diaphragm | Maintenance and size selectivity of glomerular filtration barrier. | [23] |
| NPHS2 | Slit diaphragm | Maintenance of slit diaphragm integrity | [24] |
| P-cadherin | Slit diaphragm | Stabilization of cell adherens junctions to maintain slit diaphragm integrity. | [25] |
| ZO-1 | Slit diaphragm | Maintenance of glomerular permeability by connecting slit diaphragm to actin cytoskeleton | [25] |
| Podocyte injury markers | | | |
| B7-1 (CD80) | Cell membrane (cell surface) | Upon injury *de novo* expressed causing reorganization of actin cytoskeleton and modulation of slit diaphragms. | [26] |
| CR1 | Cell membrane | During injury decreased expression whereby it may act as a protection from complement attack. | [27, 28] |
| Desmin | Foot processes (cytoskeleton) | *De novo* expressed upon injury and causes epithelial-to-mesenchymal transition. | [29, 30] |
| Ezrin | Foot processes | Injured podocytes have decreased ezrin expression that is involved in signal transduction and cell growth control. | [31, 32] |
| GLEPP-1 | Foot processes (apical membrane) | Regulation of the structure and function of foot processes, which is impaired in injured podocytes due to decreased GLEPP-1. | [33] |
| Connexin-43 | Gap junction protein | Induced expression in podocytes upon injury. | [34] |

Abbreviations: ANGPT1; angiopoietin 1, CD2AP; CD2-associated protein, MAGI1; Membrane-associated guanylate kinase, Nck ½; non-catalytic region of tyrosine kinase adaptor protein 1/ 2, NEPH1; same as kin of IRRE-like protein 1 (KIRREL), NPHS1; nephrin, NPHS2; podocin, PMAT; plasma membrane monoamine transporter, TRPC6; Transient Receptor Potential Cation Channel Subfamily C Member 6, VEGF; vascular endothelial growth factor, WT-1; Wilms tumor 1, ZO-1; zonula occludens-1, α-ACTN4; alpha actinin 4, B7-1; immune-regulatory ligand B7-1, GLEPP-1; glomerular epithelial protein 1, CR1; complement receptor 1,

# References

1. Kobayashi T, Notoya M, Shinosaki T, Kurihara H (2009) Cortactin interacts with podocalyxin and mediates morphological change of podocytes through its phosphorylation. Nephron Exp Nephrol 113:e89-96

2. Gianesello L, Priante G, Ceol M, Radu CM, Saleem MA, Simioni P, Terrin L, Anglani F, Del Prete D (2017) Albumin uptake in human podocytes: a possible role for the cubilin-amnionless (CUBAM) complex. Sci Rep 7:13705

3. Yamazaki H, Saito A, Ooi H, Kobayashi N, Mundel P, Gejyo F (2004) Differentiation-induced cultured podocytes express endocytically active megalin, a heymann nephritis antigen. Nephron Exp Nephrol 96:e52-58

4. Xia L, Zhou M, Kalhorn TF, Ho HTB, Wang J (2009) Podocyte-specific expression of organic cation transporter PMAT: implication in puromycin aminonucleoside nephrotoxicity. Am J Physiol Renal Physiol 296:F1307-1313

5. Ichimura K, Powell R, Nakamura T, Kurihara H, Sakai T, Obara T (2013) Podocalyxin regulates pronephric glomerular development in zebrafish. Physiol Rep 1:e00074

6. Suzuki K, Fukusumi Y, Yamazaki M, Kaneko H, Tsuruga K, Tanaka H, Ito E, Matsui K, Kawachi H (2015) Alteration in the podoplanin–ezrin–cytoskeleton linkage is an important initiation event of the podocyte injury in puromycin aminonucleoside nephropathy, a mimic of minimal change nephrotic syndrome. Cell Tissue Res 362:201-213

7. Yu H, Kistler A, Faridi MH, Meyer JO, Tryniszewska B, Mehta D, Yue L, Dryer S, Reiser J (2016) Synaptopodin Limits TRPC6 Podocyte Surface Expression and Attenuates Proteinuria. J Am Soc Nephrol 27:3308-3319

8. Cortes P, Mendez M, Riser BL, Guerin CJ, Rodriguez-Barbero A, Hassett C, Yee J (2000) F-actin fiber distribution in glomerular cells: structural and functional implications. Kidney Int 58:2452-2461

9. Kojima K, Davidovits A, Poczewski H, Langer B, Uchida S, Nagy-Bojarski K, Hovorka A, Sedivy R, Kerjaschki D (2004) Podocyte flattening and disorder of glomerular basement membrane are associated with splitting of dystroglycan-matrix interaction. J Am Soc Nephrol 15:2079-2089

10. Feng D, Notbohm J, Benjamin A, He S, Wang M, Ang LH, Bantawa M, Bouzid M, Del Gado E, Krishnan R, Pollak MR (2018) Disease-causing mutation in alpha-actinin-4 promotes podocyte detachment through maladaptation to periodic stretch. Proc Natl Acad Sci U S A 115:1517-1522

11. Satchell SC, Harper SJ, Tooke JE, Kerjaschki D, Saleem MA, Mathieson PW (2002) Human podocytes express angiopoietin 1, a potential regulator of glomerular vascular endothelial growth factor. J Am Soc Nephrol 13:544-550

12. Chiang WC (2013) Angiopoietins Modulate Endothelial Adaptation, Glomerular and Podocyte Hypertrophy after Uninephrectomy. PLoS ONE 8(12): e82592

13. Krendel M, Mooseker MS (2005) Myosins: tails (and heads) of functional diversity. Physiology (Bethesda) 20:239-251

14. Endlich N, Kress KR, Reiser J, Uttenweiler D, Kriz W, Mundel P, Endlich K (2001) Podocytes respond to mechanical stress in vitro. J Am Soc Nephrol 12:413-422

15. Arnemann J, Sultani O, Hasgun D, Coerdt W (2006) T-/H-cadherin (CDH13): a new marker for differentiating podocytes. Virchows Arch 448:160-164

16. Palmer RE, Kotsianti A, Cadman B, Boyd T, Gerald W, Haber DA (2001) WT1 regulates the expression of the major glomerular podocyte membrane protein Podocalyxin. Curr Biol 11:1805-1809

17. Shih NY, Li J, Cotran R, Mundel P, Miner JH, Shaw AS (2001) CD2AP localizes to the slit diaphragm and binds to nephrin via a novel C-terminal domain. Am J Pathol 159:2303-2308

18. Yaoita E, Kurihara H, Yoshida Y, Inoue T, Matsuki A, Sakai T, Yamamoto T (2005) Role of Fat1 in cell-cell contact formation of podocytes in puromycin aminonucleoside nephrosis and neonatal kidney. Kidney Int 68:542-551

19. Ni J, Bao S, Johnson RI, Zhu B, Li J, Vadaparampil J, Smith CM, Campbell KN, Grahammer F, Huber TB, He JC, D'Agati VD, Chan A, Kaufman L (2016) MAGI-1 Interacts with Nephrin to Maintain Slit Diaphragm Structure through Enhanced Rap1 Activation in Podocytes. J Biol Chem 291:24406-24417.

20. Verma R, Kovari I, Soofi A, Nihalani D, Patrie K, Holzman LB (2006) Nephrin ectodomain engagement results in Src kinase activation, nephrin phosphorylation, Nck recruitment, and actin polymerization. J Clin Invest 116:1346-1359

21. Li H, Zhu J, Aoudjit L, Latreille M, Kawachi H, Larose L, Takano T (2006) Rat nephrin modulates cell morphology via the adaptor protein Nck. Biochem Biophys Res Commun 349:310-316

22. Liu G, Kaw B, Kurfis J, Rahmanuddin S, Kanwar YS, Chugh SS (2003) Neph1 and nephrin interaction in the slit diaphragm is an important determinant of glomerular permeability. J Clin Invest 112:209-221

23. Welsh GI, Saleem MA (2010) Nephrin-signature molecule of the glomerular podocyte? J Pathol 220:328-337

24. Roselli S, Gribouval O, Boute N, Sich M, Benessy F, Attié T, Gubler MC, Antignac C (2002) Podocin Localizes in the Kidney to the Slit Diaphragm Area. Am J Pathol 160:131-139

25. Reiser J, Kriz W, Kretzler M, Mundel P (2000) The glomerular slit diaphragm is a modified adherens junction. J Am Soc Nephrol 11:1-8

26. Reiser J, von Gersdorff G, Loos M, Oh J, Asanuma K, Giardino L, Rastaldi MP, Calvaresi N, Watanabe H, Schwarz K, Faul C, Kretzler M, Davidson A, Sugimoto H, Kalluri R, Sharpe AH, Kreidberg JA, Mundel P (2004) Induction of B7-1 in podocytes is associated with nephrotic syndrome. J Clin Invest 113:1390-1397

27. Ichida S, Yuzawa Y, Okada H, Yoshioka K, Matsuo S (1994) Localization of the complement regulatory proteins in the normal human kidney. Kidney Int 46:89-96

28. Moll S, Miot S, Sadallah S, Gudat F, Mihatsch MJ, Schifferli JA (2001) No complement receptor 1 stumps on podocytes in human glomerulopathies. Kidney Int 59:160-168

29. Zou J, Yaoita E, Watanabe Y, Yoshida Y, Nameta M, Li H, Qu Z, Yamamoto T (2006) Upregulation of nestin, vimentin, and desmin in rat podocytes in response to injury. Virchows Arch 448:485-492

30. Matsusaka T, Sandgren E, Shintani A, Kon V, Pastan I, Fogo AB, Ichikawa I (2011) Podocyte Injury Damages Other Podocytes. J Am Soc Nephrol 22:1275-1285

31. Celik H, Bulut G, Han J, Graham GT, Minas TZ, Conn EJ, Hong SH, Pauly GT, Hayran M, Li X, Ozdemirli M, Ayhan A, Rudek MA, Toretsky JA, Uren A (2016) Ezrin Inhibition Up-regulates Stress Response Gene Expression. J Biol Chem 291:13257-13270

32. Ostalska‐Nowicka D, Zachwieja J, Nowicki M, Kaczmarek E, Siwinska A, Witt M (2006) Ezrin—a useful factor in the prognosis of nephrotic syndrome in children: an immunohistochemical approach. J Clin Pathol 59:916-920

33. Tian J, Wang HP, Mao YY, Jin J, Chen JH (2007) Reduced glomerular epithelial protein 1 expression and podocyte injury in immunoglobulin A nephropathy. J Int Med Res 35:338-345

34. Yaoita E, Yao J, Yoshida Y, Morioka T, Nameta M, Takata T, Kamiie J, Fujinaka H, Oite T, Yamamoto T (2002) Up-regulation of connexin43 in glomerular podocytes in response to injury. Am J Pathol 161:1597-1606
